# MicroRNAs in cancers and neurodegenerative disorders

**DOI:** 10.3389/fgene.2012.00194

**Published:** 2012-09-26

**Authors:** Yoshimasa Saito, Hidetsugu Saito

**Affiliations:** Division of Pharmacotherapeutics, Keio University Faculty of PharmacyTokyo, Japan

**Keywords:** microRNA, cancer, neurodegenerative disorder, *miR-9*, *miR-29a/29b-1*, *miR-34b/34c*

## Abstract

MicroRNAs (miRNAs) are small non-coding RNAs which function as endogenous silencers of various target genes. miRNAs are expressed in a tissue-specific manner and playing important roles in cell proliferation, apoptosis, and differentiation during mammalian development. Links between miRNAs and the initiation and progression of human diseases including cancer are becoming increasingly apparent. Recent studies have revealed that some miRNAs such as *miR-9*, *miR-29* family, and *miR-34* family are differentially expressed in Alzheimer’s disease, Parkinson’s disease, and Huntington’s disease. These miRNAs are also reported to act as tumor suppressors during human carcinogenesis. In this review, we discuss about miRNAs which are important in the molecular pathogenesis of both cancer and neurodegeneration. Cancer and neurodegenerative disorder may be influenced by common miRNA pathways that regulate differentiation, proliferation, and death of cells.

## THE BIOGENESIS OF miRNA

**FIGURE 1:**
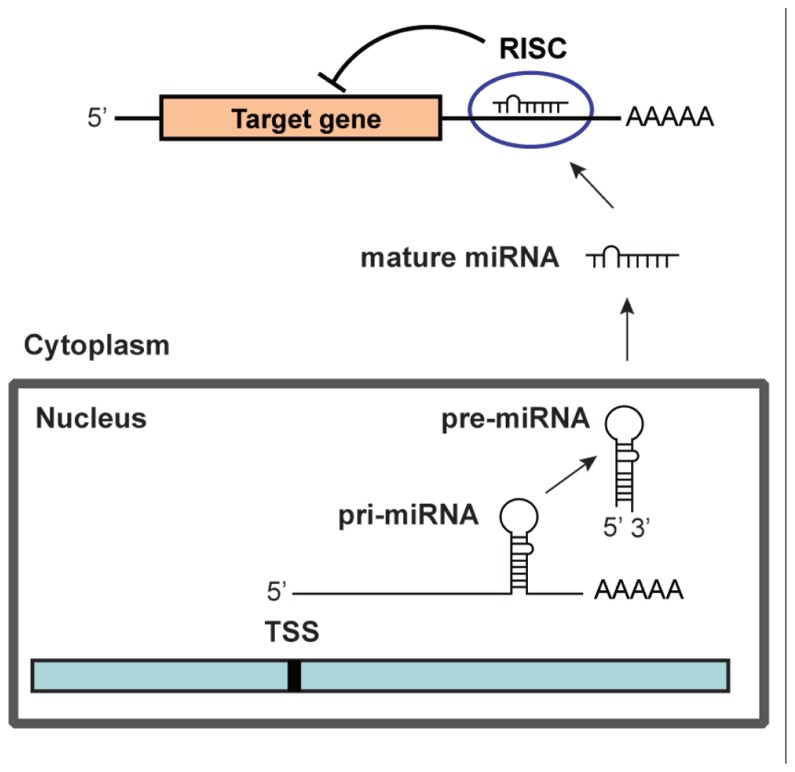
** The biogenesis of miRNA**.

MicroRNAs (miRNAs) are ~22 nucleotide (nt) non-coding RNAs that can post-transcriptionally downregulate the expression of various target genes. Currently, ~1500 human miRNAs have been identified in the human genome, each of which potentially controls hundreds of target genes. As shown in **Figure 1**, miRNA genes are generally transcribed from transcription start sites (TSSs) by RNA polymerase II (pol II) to form primary transcripts (pri-miRNAs). Pol II-transcribed pri-miRNAs are capped with 7-methylguanosine and are polyadenylated. The nuclear RNase III enzyme Drosha and its co-factor DGCR8 process pri-miRNAs into ~60-nt precursor miRNAs (pre-miRNAs), which form an imperfect stem-loop structure. Pre-miRNAs are transported into the cytoplasm by exportin 5 and are subsequently cleaved by Dicer into mature miRNAs, which are then loaded into the RNA-induced silencing complex (RISC). The miRNA/RISC complex downregulates specific gene products by translational repression via binding to partially complementary sequences in the 3′-untranslated regions (UTRs) of the target mRNAs or by directing mRNA degradation via binding to perfectly complementary sequences (He and Hannon, 2004). One strand of the miRNA duplex, which is derived from pre-miRNA, remains stably associated with RISC and guides it mainly, but not exclusively, to the 3′-UTR of the target mRNAs. Targeting can also occur in the 5′-UTR of the target mRNAs (Lewis et al., 2005; Lytle et al., 2007; Qin et al., 2010). In addition, a recent study has revealed that miRNAs are not only able to downregulate specific gene products but also can activate the expression of a target gene (Vasudevan et al., 2007). miRNAs are expressed in a tissue-specific manner and play important roles in cell proliferation, apoptosis, and differentiation during mammalian development. Links between miRNAs and the development of human malignancies are becoming increasingly apparent (Calin and Croce, 2006, 2007; Saito et al., 2009a).

## miRNA AS TUMOR SUPPRESSORS AND ONCOGENES

The finding that predicted targets of miRNAs are enriched for genes involved in transcriptional regulation, cell proliferation, and apoptosis implies that aberrant expression of miRNAs might contribute to the initiation and progression of human cancer. Onco-miRNAs and suppressor-miRNAs can represent two different looks of the same gene, behaving as oncogenes or tumor suppressors depending on tissue type and specific targets (Fabbri et al., 2007b). Calin et al. (2004) examined the mapping of 186 human miRNAs and compared their locations to genomic regions involved in cancers. They found that miRNA genes are frequently located at fragile sites which include loss of heterozygosity (LOH), amplification, and breakpoints, indicating that these miRNAs may be dysregulated in human cancers. Some miRNAs are downregulated in various human cancers, which suggests that they may function as tumor suppressors. *let-7* is downregulated in lung cancer and targets a critical oncogene *RAS* (Johnson et al., 2005). *miR-15* and *miR-16* are downregulated in chronic lymphocytic leukemia and target an antiapoptotic gene *BCL2* (Cimmino et al., 2005). *miR-143* and *miR-145* are downregulated in colorectal and breast cancer (Michael et al., 2003; Iorio et al., 2005).

On the other hand, some miRNA genes are overexpressed in cancers, indicating that they may have roles as oncogenes and accelerate the development of cancer. *miR-155* and its host gene *BIC* are highly expressed in several types of B cell lymphoma (Eis et al., 2005). High expression of precursor *miR-155* is reported in children with Burkitt lymphoma (Metzler et al., 2004). Moreover, recent studies have shown that *miR-155* is overexpressed in several types of human solid tumors including breast, colon, and lung cancer (Iorio et al., 2005; Volinia et al., 2006; Yanaihara et al., 2006). These studies strongly suggest that *miR-155* is an oncogenic miRNA. The *miR-17-92* cluster, which is located on chromosome 13q31, is activated by the oncogene *c-Myc* which is an important regulator of cell growth and is often mutated or amplified in human cancers. The *miR-17-92* cluster is highly expressed in breast, colon, lung, pancreatic, and prostate cancer, as well as in B cell lymphoma (Hayashita et al., 2005; He et al., 2005; Volinia et al., 2006). c-Myc binding upregulates the transcription factor *E2F1* and two miRNAs in the *miR-17-92* cluster, *miR-17-5p* and *miR-20a*, target *E2F1* (O’Donnell et al., 2005). This suggests that there is a negative feedback loop involving *c-Myc*, *E2F1*, *miR-17-5p*, and *miR-20a* whereby c-Myc induces expression of *E2F1* and the post-transcriptional repressors of *E2F1*, *miR-17-5p*, and *miR-20a*. Volinia et al. (2006) showed that *miR-20a* also targets the tumor suppressor transforming growth factor beta receptor 2 (*TGFBR2*). Chan et al. (2005) showed that *miR-21* is strongly overexpressed in the highly malignant brain tumor glioblastoma. They also revealed that knockdown of *miR-21* in cultured glioblastoma cells induces activation of caspases and leads to increased apoptotic cell death, suggesting that *miR-21* is an antiapoptotic factor in human glioblastoma cells. *miR-21* is upregulated in breast, colon, lung, pancreatic, prostate, and stomach cancer (Iorio et al., 2005; Volinia et al., 2006). Moreover, *miR-21* is highly overexpressed in human cholangiocarcinoma and modulates gemcitabine-induced apoptosis by directly altering *PTEN* expression (Meng et al., 2007). Taken together, these findings indicate that miRNAs have critical roles in the mechanism of human carcinogenesis and that aberrant expression of miRNAs may contribute to the development of human cancer.

## miRNA IN CANCERS AND NEURODEGENERATIVE DISORDERS

Cancer and neurodegenerative disease may be influenced by common signaling pathways regulating the balance of cell survival and death. Recent studies suggest that the molecular machinery involved in maintaining neural function in neurodegenerative disease may be shared with oncogenic pathways. p53 is one of the most extensively studied proteins because of its role in cancer prevention, and has been recently shown to be involved in aging and Alzheimer’s disease (AD). The observation that aging and AD interfere with proteins controlling duplication and cell cycle may lead to the speculation that, in senescent neurons, aberrations in proteins generally dealing with cell cycle control and apoptosis could affect neuronal plasticity and functioning rather than cell duplication (Lanni et al., 2012).

Recent studies have revealed that some miRNAs are aberrantly expressed in the brains of patients with neurodegenerative diseases such as AD, Parkinson’s disease (PD) and Huntington’s disease (HD; Junn and Mouradian, 2012). This suggests that miRNAs play critical roles in neurodegeneration as well as cancer. Specific miRNAs may regulate the expression of their target proteins that are involved in the development of neurodegenerative diseases. Interestingly, several miRNAs differentially expressed in neurodegenerative diseases such as *miR-9*, *miR-29* family, and *miR-34* family are considered to be potential tumor suppressor miRNAs (**Table 1**). Cancer and neurodegenerative disorder may be influenced by common miRNA pathways that regulate differentiation, proliferation and death of cells.

**Table 1:** miRNAs differentially expressed in cancers and neurodegenerative disorders.

miRNA	Expression in cancers	Expression in neurodegenerative disorders	Target genes	Reference
*miR-9*	Silenced in breast cancer and cancer metastasis by DNA methylation	Decreased in Huntington’s disease	*REST/CoREST*	Chan et al. (2005), O’Donnell et al. (2005),Lanni et al. (2012)
*miR-29a/29b-1*	Decreased in various cancers	Decreased in Alzheimer’s disease	*MCL1, DNMT3A, DNMT3B, BACE1*	Lujambio et al. (2008),Du et al. (2012),Junn and Mouradian (2012)
*miR-34b/34c*	Silenced in colon cancer and cancer metastasis by DNA methylation	Decreased in Parkinson’s disease	*MET, CCNE2, CDK4, CDK6*	Fabbri et al. (2007a), Mott et al. (2007)

### miR-9

Aberrant DNA methylation at CpG island promoters of tumor suppressor genes is one of the most important mechanisms of human carcinogenesis. Recent studies have shown that *miR-9* is silenced by aberrant CpG island methylation in various human cancers including breast cancer, cancer metastasis, gastric cancers, and lung cancers, suggesting that *miR-9* is a potential tumor suppressor miRNA (Lehmann et al., 2008; Lujambio et al., 2008; Du et al., 2012; Heller et al., 2012).

Huntington’s disease is an autosomal dominant neurodegenerative disease caused by CAG trinucleotide repeat expansion in *huntingtin*, which encodes Huntingtin (Htt). Patients with HD experience abnormal motor movements, cognitive decline, and psychiatric disturbances that frequently result in premature death. Although Htt is ubiquitously expressed, patients with HD show predominantly CNS manifestations. One putative mechanism underlying the transcriptional changes is aberrant cellular distribution of the transcriptional repressor RE1-silencing transcription factor (REST). REST expression is highest in pluripotent stem cells and decreases upon restriction to neural progenitor cells and subsequently to neurons. REST silences neuronal gene expression in non-neuronal cells. In mature, healthy neurons, REST is expressed at low levels and primarily sequestered in the cytoplasm in part through interaction with Htt. However, in patients with HD, mutant Htt fails to bind REST, enabling its nuclear translocation. In the nucleus, REST can bind RE1 consensus sequences and recruit corepressors including mSin3, REST corepressor 1 (CoREST), and methyl CpG binding protein 2 (MeCP2) to inactivate neuron-specific genes (Zuccato et al., 2003).

Packer et al. (2008) have reported that levels of several miRNAs with upstream RE1 sites are decreased in HD patient cortices relative to healthy controls. One of these, *miR-9* and miR-9*, which decreased early in HD, are processed from the same primary transcript from three genomic loci (*miR-9-1*, *miR-9-2*, and *miR-9-3*). *miR-9-1* and *miR-9-3* both have upstream RE1 sequences that can be occupied by REST. Interestingly, miR-9/miR-9*, targets two components of the REST complex: *miR-9* targets REST and miR-9* targets CoREST. These data provide evidence for a double negative feedback loop between the REST silencing complex and miR-9/miR-9* (Packer et al., 2008).

### miR-29 FAMILY

A recent study has revealed that *miR-29* family (*miR-29a*, *miR-29b*, and *miR-29c*) target the *de novo* DNA methyltransferases *DNMT3A* and *DNMT3B* and expression levels of *miR-29* family were suppressed in lung cancer. The reduced expression of the *miR-29* family induced overexpression of *DNMT3A* and *DNMT3B*, resulting in aberrant DNA methylation in lung cancer (Fabbri et al., 2007a). In addition, Mott et al. (2007) have demonstrated that *MCL1*, encoding an antiapoptotic BCL2 family protein, is one of the targets of *miR-29* family, and that *miR-29* miRNAs regulate apoptosis by targeting *MCL1*. These findings suggest that *miR-29* family act as tumor suppressors by targeting *DNMT3A*, *DNMT3B*, and* MCL1*.

Mutations in the *APP* and *PSEN* genes cause amyloid β (Aβ) accumulation and familial AD. However, little is known about the mechanisms that contribute to Aβ accumulation in the vast majority of sporadic AD cases. BACE1/β-secretase cleavage of APP is the rate-limiting step for Aβ peptide production. Increased BACE1 expression is observed in patients with sporadic AD, and several mechanisms for this upregulation have been proposed. A link between BACE1 levels, Aβ load, and AD pathology has been reported, suggesting that increased BACE1 expression is indeed an important risk factor for sporadic AD (Li et al., 2004).

Hebert et al. (2008) investigated changes in miRNA expression profiles of sporadic AD patients and found that several miRNAs potentially involved in the regulation of *APP* and *BACE1* expression appeared to be decreased in their brain. They have shown that *miR-29a*, *miR-29b-1*, and *miR-9* can regulate *BACE1* expression as their targets. The *miR-29a/b-1* cluster was significantly decreased in AD patients displaying overexpression of BACE1 protein. Similar correlations between expression of this cluster and BACE1 were found during brain development and in primary neuronal cultures. They provided evidence for a potential causal relationship between *miR-29a/b-1* expression and Aβ generation in a cell culture model and proposed that loss of specific miRNAs can contribute to increased BACE1 and Aβ levels in sporadic AD (Hebert et al., 2008). A recent study has shown that *miR-29c* also regulates BACE1 protein expression (Zong et al., 2011). These findings suggest that *miR-29* family regulate *BACE1* expression and play important roles in the pathogenesis of AD.

### miR-34 b/34c

*miR-34a* was identified as a target of p53 and induces a G (He and Hannon, 2004) cell cycle arrest, senescence, and apoptosis (He et al., 2007; Tazawa et al., 2007). *miR-34a* expression is silenced in several types of cancer including pancreatic cancer due to aberrant CpG methylation of its promoter. Re-expression of *miR-34a* in a pancreatic cancer cell line induced senescence and cell cycle arrest by targeting *CDK6*, indicating that *miR-34a* represents a tumor suppressor gene which is inactivated by CpG methylation in pancreatic cancer (Lodygin et al., 2008). *miR-34b* and *miR-34c* are also reported to be direct targets of p53 and silenced by aberrant CpG island methylation in colorectal cancer (Toyota et al., 2008).

Parkinson’s disease is the second most common neurodegenerative disorder, characterized by the presence of protein inclusions or Lewy bodies and a progressive loss of dopaminergic neurons in the midbrain. Minones-Moyano et al. (2011) have evaluated miRNA expression deregulation in PD brain samples. miRNA expression profiles revealed decreased expression of *miR-34b* and *miR-34c* in brain areas with variable neuropathological affectation at clinical stages of PD. Downregulation of *miR-34b/c* was detected in pre-motor stages of PD. Depletion of *miR-34b* or *miR-34c* in differentiated SH-SY5Y dopaminergic neuronal cells resulted in a moderate reduction in cell viability that was accompanied by altered mitochondrial function and dynamics, oxidative stress and reduction in total cellular adenosine triphosphate content. Moreover, they have shown that DJ1 and Parkin are indirect targets of *miR-34b/c*. *miR-34b/c* downregulation induced a decrease in the expression of DJ1 and Parkin, two proteins associated to familial forms of PD. DJ1 and Parkin expression was reduced in PD brain samples displaying strong *miR-34b/c* downregulation. These data suggest that early deregulation of *miR-34b/c*, which are direct targets of p53, in PD triggers downstream transcriptome alterations underlying mitochondrial dysfunction and oxidative stress, which ultimately compromise cell viability (Minones-Moyano et al., 2011). As mentioned above, p53 is also involved in the pathogenesis of AD. These findings indicate that p53 may play important roles in the initiation and progression of both AD and PD via different miRNA-mediated mechanisms.

## miRNA-MEDIATED THERAPY FOR CANCER AND NEURODEGENERATIVE DISEASE

The distinct connection between aberrant expression of miRNAs and human diseases suggests that miRNAs could be therapeutic targets. Epigenetic changes such as DNA methylation and histone modification modulate chromatin structure and gene expression in mammalian development and in human diseases (Egger et al., 2004). Many miRNAs are expressed in a tissue- and tumor-specific manner, implying that some miRNAs are subject to epigenetic control. We have recently shown that ~5% of human miRNAs are upregulated more than threefold by treatment of T24 bladder cancer cells with the DNA demethylating agent 5-aza-2′-deoxycytidine (5-Aza-CdR) and the histone deacetylase (HDAC) inhibitor 4-phenylbutyric acid (PBA). In particular, *miR-127*, which is embedded in a CpG island, is remarkably induced by a decrease in DNA methylation levels and an increase in active histone marks around the promoter region of the *miR-127* gene. In addition, activation of *miR-127* by epigenetic treatment induced downregulation of its target oncogene *BCL6* (Saito et al., 2006). We have also demonstrated that treatment of gastric cancer cells with 5-Aza-CdR and PBA induces activation of *miR-512-5p* which is located at Alu repeats on chromosome 19. Activation of *miR-512-5p* by epigenetic treatment induces suppression of *MCL1*, resulting in apoptosis of gastric cancer cells (Saito et al., 2009b). These results indicate that chromatin remodeling by epigenetic treatment can directly modulate expression of miRNAs that are involved in the pathogenesis of cancer and neurodegenerative disease. In addition, recent study has shown that locked-nucleic-acid-modified oligonucleotide (LNA-antimiR) effectively antagonizes specific miRNAs and could be used to silence miRNAs that are overexpressed in human diseases (Elmen et al., 2008). Because the link between miRNAs and cancer and neurodegeneration has only just begun to be understood, there could be a large number of critical miRNAs and their target genes. Further studies are necessary to identify the specific miRNAs that could be therapeutic targets and/or molecular markers for human cancers and neurodegenerative disorders.

## Conflict of Interest Statement:

The authors declare that the research was conducted in the absence of any commercial or financial relationships that could be construed as a potential conflict of interest.
